# Interaction of Polystyrene Nanoplastics with Biomolecules and Environmental Pollutants: Effects on Human Hepatocytes †

**DOI:** 10.3390/ijms26072899

**Published:** 2025-03-22

**Authors:** Barbara Mognetti, Claudio Cecone, Katia Fancello, Astrid Saraceni, Erika Cottone, Patrizia Bovolin

**Affiliations:** 1 Department of Life Sciences and Systems Biology, University of Turin, Via Accademia Albertina 13, 10123 Turin, Italy; katia.fancello@edu.unito.it (K.F.); astrid.saraceni@unito.it (A.S.); erika.cottone@unito.it (E.C.); patrizia.bovolin@unito.it (P.B.); 2 SUSPLAS@UniTo, Sustainable Plastic Scientific Hub, University of Turin, 10100 Turin, Italy; claudio.cecone@unito.it; 3 Department of Chemistry, University of Turin, Via Pietro Giuria 7, 10125 Turin, Italy

**Keywords:** nanoplastics, cadmium, hepatocyte viability, oxidative stress, lipid sequestration

## Abstract

The inevitable exposure of humans to micro/nanoplastics has become a pressing global environmental issue, with growing concerns regarding their impact on health. While the direct effects of micro/nanoplastics on human health remain largely unknown, increasing attention is being given to their potential role as carriers of environmental pollutants and organic substances. This study investigates the direct toxicity of 500 nm polystyrene nanoplastics (NPs) on human hepatocytes (HepG2) in vitro, both alone and in combination with cadmium (Cd), a hazardous heavy metal and a prevalent environmental pollutant. One-hour exposure to 100 µg/mL of NPs causes a significant increase in ROS production (+25% compared to control) but cell viability remains unaffected even at concentrations much higher than environmental levels. Interestingly, NPs significantly reduce Cd cytotoxicity at LC_50_ concentrations (cell viability compared to control: 55.4% for 50 µM Cd, 66.9% for 50 µM Cd + 10 µg/mL NPs, 68.4% for 50 µM Cd + 100 µg/mL NPs). Additionally, NPs do not alter the cellular lipid content after short-term exposure (24 h). However, when Cd and fatty acids are added to the medium, NPs appear to sequester fatty acids, reducing their availability and impairing their uptake by cells in a dose-dependent manner. We confirmed by Dynamic Light Scattering and Scanning Electron Microscopy the interaction between NPs, Cd and free fatty acids. Although polystyrene NPs exhibited minimal cytotoxicity in our experimental model, collectively our findings suggest that predicting the effects of cell exposure to NPs is extremely challenging, due to the potential interaction between NPs, environmental pollutants and specific components of the biological matrix.

## 1. Introduction

The global issue of microplastic pollution in water, air and soil is becoming a growing environmental challenge, whose potential impact on human health is still far from being understood. Microplastics (MPs), measuring 1 µm to 5 mm in size, and nanoplastics (NPs), ranging from 1 to 1000 nm in size [1,2], are plastic particles intentionally produced for use in cosmetics, as drug carriers, and for applications in various other industries. Alternatively, they can originate from the decomposition of larger plastics items due to complex environmental factors (e.g., physical abrasion, UV radiation, waves, wind, temperature, etc.). This implies that the nature of MPs and NPs dispersed in the environment is highly variable, encompassing a wide range of sizes, compositions, and shapes. 

Human exposure to MPs/NPs primarily occurs through inhalation of indoor air [3,4], consumption of drinking water [5], and ingestion within the food chain [3]. While larger plastic polymers are generally considered inert and not absorbed in the intestinal system, smaller particles might undergo intestinal absorption, even though most are excreted in feces [6]. Data on NP and MP absorption in humans is still limited. Although there is no definitive evidence demonstrating the translocation of MPs and NPs across the intestinal barrier in human, evidence from in vitro models supports this hypothesis [7,8,9,10]. In particular, smaller particles (20–100 nm in size) are primarily translocated by enterocytes [7,8,9,10], while larger ones (up to 10 μm) are mainly translocated by microfold cells (M cells) associated with Peyer’s patches [7,11,12,13]. According to Schwarzfischer and Rogler [14], it is plausible that microplastics uptake might follow mechanisms identified for TiO_2_ microparticles, including paracellular transport across tight junctions, transcytosis via M-cells in Peyer’s patches, and persorption through degrading enterocytes. The distribution to various organs of particles absorbed at the intestinal level has been reported in studies conducted on mice [15,16] and zebrafish [17]. Indeed, MPs have been found in human organs and tissues including colectomy specimens, blood and liver (rev. in [18]).

Studies using in vivo models have delineated various effects on the liver, notably the presence of MPs in the hepatic parenchyma [19], accumulation of fatty acids and induction of metabolic disorders [20], elevation of bile acids and their metabolites and the manifestation of intestinal dysbiosis [21]. Moreover, mice exposed to microplastics showed impaired glucose tolerance and hepatic lipid deposition in response to high-dose microplastics administration [22]. Conversely, when 42 nm nanoplastics were administered intravenously into the tails of high-fat-diet-fed mice, inflammatory response was up-regulated in the liver, as evidenced by enhanced infiltration of Kupffer cells and elevated expression of pro-inflammatory markers [23]. In an in vitro 3D model of human pluripotent stem cells-derived liver organoids, microplastics caused hepatotoxicity and disruption of lipid metabolism [24].

Beyond the direct impact of MPs on organisms, research and discussions have focused on their role as vectors transporting other environmental pollutants into organisms [25]. Due to their significant surface area, MPs have the capacity to readily adsorb and concentrate environmental pollutants, including heavy metals and organic contaminants [26,27]. The concurrent presence of MPs and environmental pollutants may constitute potential hazards to both human health and the ecosystem. Earlier investigations have revealed that the interactions between MPs and other environmental contaminants can significantly alter their ambient dynamics, toxicity, and bioavailability [28,29,30]. Recent studies have shown that microplastics can adsorb and accumulate hazardous organic pollutants from aquatic, air and soil systems. This leads to concentrations of organic pollutants carried by the microplastics that are hundreds or thousands of times higher than those found in the surrounding environment [31]. The same may apply to inorganic pollutants, namely heavy metals, which can adhere to microplastics due to their surface properties, potentially leading to increased toxicity [32,33,34]. In this context, nanoplastics deserve even greater attention, as their smaller size and larger surface area-to-volume ratio compared to MPs may increase their adsorption capacity for environmental substances [35].

As highlighted by some authors in recent publications [36,37] it is unrealistic to consider the effects of MPs/NPs without taking into account the interactions they may have with biological or environmental molecules. Given the complexity of this environmental issue [37], we opted to test the biological impact of NPs in an in vitro model. The goal was to assess their acute toxicity and effects on lipid content on a hepatocarcinoma cell line. The choice of hepatocytes is motivated by their potential exposure to high concentrations of ingested NPs absorbed at the intestinal level. To study the interplay between NPs and environmental pollutants, we focused on cadmium (Cd), a heavy metal and widespread contaminant that primarily affects the liver and kidneys by inducing oxidative stress, mitochondrial damage, and inflammation [38,39]. Cd is not only a prevalent environmental pollutant but is also found within the human body; accumulated amounts range from 0.14 to 3.2 ppm in muscles (1.25 × 10^−6^ M–2.85 × 10^−5^ M), 1.8 ppm in bones (1.6 × 10^−5^ M), and 0.0052 ppm in the blood (4.63 × 10^−8^ M) [40,41]. Using a combination of physico-chemical and biological tests, we therefore examined the interactions between Cd, polystyrene NPs and cell medium components, investigating their potential synergistic effects on cell survival and metabolism.

## 2. Results

### 2.1. Polystyrene NPs Do Not Affect HepG2 Viability

**Figure 1:**
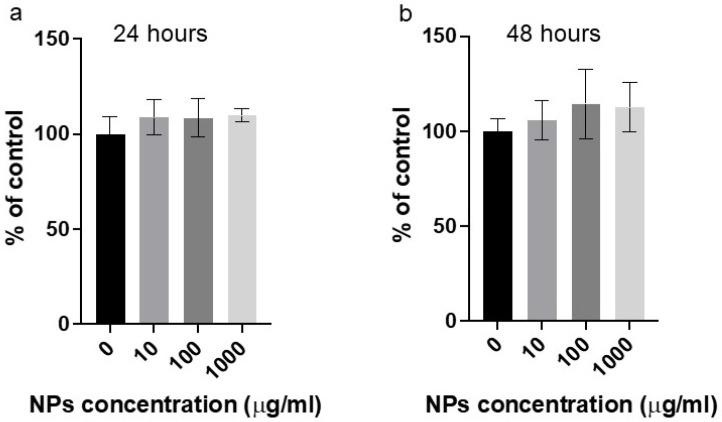
The effects of increasing concentrations of nanoparticles (NPs) on cell viability, as measured by the CellTiter-Glo^®^ luminescent Cell Viability Assay after (**a**,**b**) 24 or 48 hours of exposure to NPs. Cell viability is expressed as luminescence intensity, which is directly proportional to the number of viable cells. Each graph represents the mean of three independent experiments (n = 3) ± standard deviation (SD).

In order to determine the direct cytotoxicity of nanoplastics, HepG2 cells were exposed to increasing concentrations of NPs and, at the end of the incubation period, cell viability was tested. The results presented in Figure 1 show that NPs (10–1000 µg/mL) do not induce significant cytotoxicity in cells, even at concentrations far exceeding typical environmental levels. 

### 2.2. Polystyrene NPs Mitigate Cadmium Toxicity Possibly Through Sequestration

To investigate whether the presence of NPs might interact with cadmium cytotoxicity, we incubated the cells with cadmium alone and with both cadmium and NPs. 

**Figure 2:**
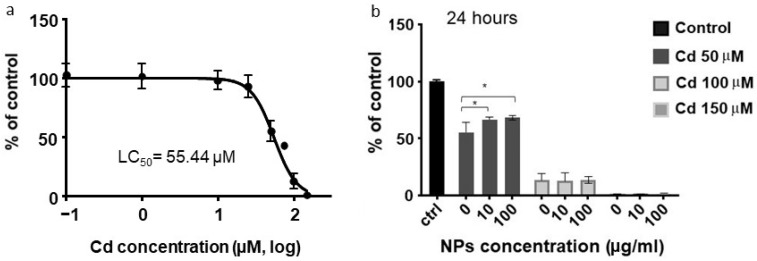
The effects of cadmium (Cd) alone (panel **a**) and in combination with two different concentrations of nanoparticles (NPs) (panel **b**) on cell viability, as determined by the CellTiter-Glo^®^ luminescent Cell Viability Assay. Cell viability is quantified based on luminescence intensity, which is proportional to the number of living cells. The LC_50_ (lethal concentration 50) of Cd is indicated in panel (**a**). Each graph depicts the mean results of three independent experiments (n = 3) with error bars representing the standard deviation (SD). * *p* < 0.05.

As expected, Cd displayed a dose-dependent cytotoxic effect, with a LC_50_ value of 55.44 µM (Figure 2a). Interestingly, the co-exposure of cells to 10–100 µg/mL NPs and 50 µM Cd (a concentration close to the LC_50_ value) resulted in a significant reduction in cadmium-induced toxicity (Figure 2b, *p* < 0.05).

### 2.3. Non Cytotoxic NPs Concentrations Significantly Increase ROS Production in HepG2 Cells

**Figure 3:**
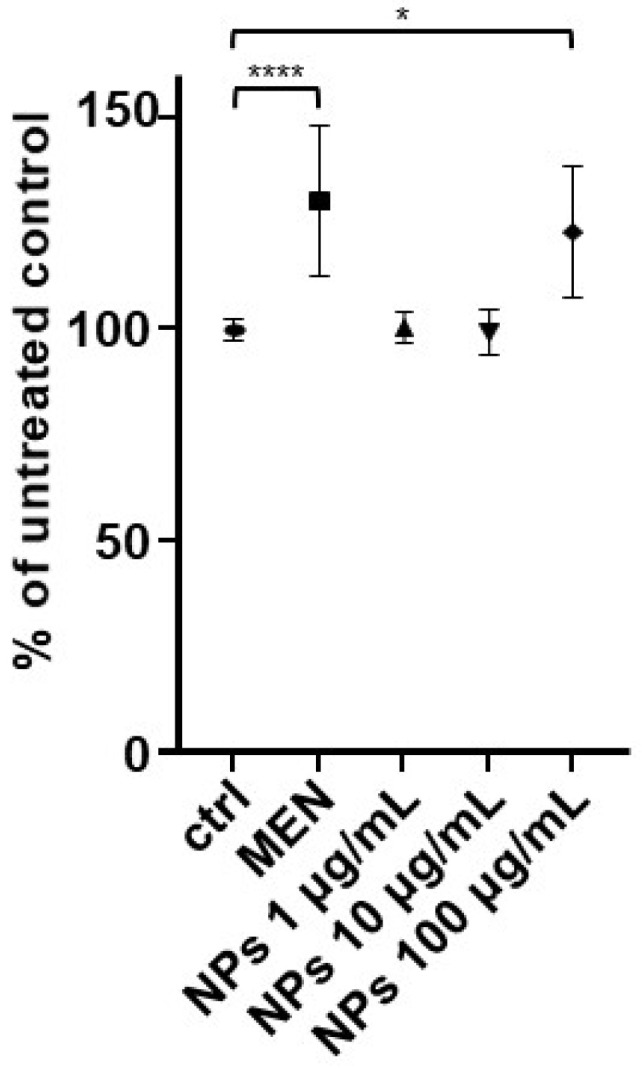
Production of reactive oxygen species (ROS) after exposing cells to 20 µM of MEN or different concentrations of nanoparticles (NPs) for 1 h. After exposure, cells were loaded with the CellROX^®^ Green probe, which fluoresces upon oxidation by ROS, allowing for quantification of ROS production. Fluorescence was recorded using a microplate reader. The data, expressed as a percentage relative to the control condition, represent the mean ± standard deviation (SD) of three independent experiments (n = 3) * *p* < 0.05; **** *p* < 0.001.

In order to verify whether brief exposure to NPs induces the production of reactive oxygen species (ROS) in HepG2 cells, highlighting potential oxidative stress, cells were exposed to different concentrations of NPs (1 µg/mL, 10 µg/mL and 100 µg/mL), or to 20 µM menadione (MEN), a known ROS inducer, for 1 h. The untreated control (ctrl) group was set to 100%. As shown in Figure 3, menadione alone significantly increased ROS production (**** *p* < 0.0001). ROS levels remained similar to the control at low and intermediate NP concentrations (1 µg/mL and 10 µg/mL). On the other hand, ROS production became significantly higher at 100 µg/mL of NPs (* *p* < 0.05), suggesting that this concentration triggers a notable increase in oxidative stress. 

### 2.4. NPs Do Not Affect Endogenous Lipid Content or Synthesis but Reduce Cellular Lipid Uptake in the Presence of Cd

**Figure 4:**
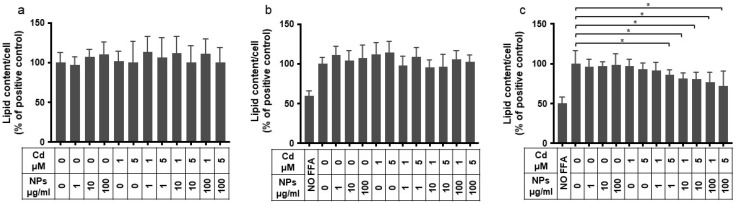
Lipid content quantification in HepG2 cells following exposure to NPs, Cd, or a combination of both. (**a**) Cells were exposed for 24 h to NPs, Cd, or NPs + Cd in standard culture conditions. (**b**) Following a 24 h starvation period, cells were grown in a medium containing a mixture of fatty acids (FFA, 0.5 mM) for 24 h. After this period, FFA were removed, and cells were then exposed to NPs, Cd, or NPs + Cd. (**c**) After 24 h starvation, cells were exposed for 24 h to FFA together with NPs, Cd, or NPs + Cd. At the end of each experiment, the AdipoRed/NucBlue (A/N) assay was performed for lipid quantification. The data displayed in all three panels are expressed as percentages of the control condition and are represented as mean ± standard deviation (SD). Statistical significance is indicated by asterisks: * *p* < 0.05.

The aim of this experiment was to verify whether exposing HepG2 to NPs affects their lipid content, both basal or induced by the internalization of free fatty acids added to the medium. Additionally, we wanted to see if the simultaneous presence of Cd in the culture medium would generate an interaction with the aforementioned processes. HepG2 cells were exposed to various non cytotoxic concentrations of NPs (Figure 1) and Cd (Figure 2a) for 24 h to assess their effects on the endogenous lipid content (the experimental design is outlined in Section 4, Table 1). Figure 4a shows that no changes in cellular lipid content were induced from either pollutant, whether individually or in combination. Similar results were observed in cells in which lipid accumulation was induced by 24 h incubation with a free fatty acid mixture (FFA), and subsequently exposed to NPs and Cd, either alone or in combination (Figure 4b). In contrast, when cells were exposed simultaneously to FFA and both Cd and NPs, a significant reduction in the intracellular lipid content was observed. The effect was not seen when exposing cells to FFA and Cd or FFA and NPs alone (Figure 4c).

### 2.5. NPs Characterization and Their Interactions with Cd and FFA

**Figure 5:**
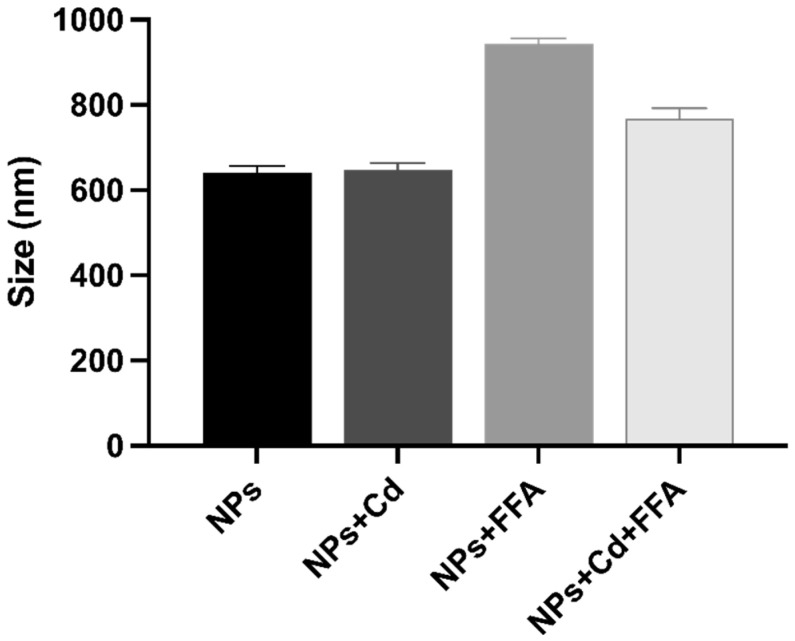
Hydrodynamic diameters of nanoparticles (NPs) measured using Dynamic Light Scattering (DLS). NPs were dispersed in Minimum Essential Medium with or without cadmium (Cd) and free fatty acids (FFA), following the same procedure used for the in vitro tests. For each sample, triplicate measurements were performed, with each measurement averaging at least 10 runs. The data shown represent the mean hydrodynamic diameters ± standard deviation (SD).

To understand the behavior of nanoplastics in our in vitro model, particularly their potential interactions with other components of the experimental system, we conducted both physical and morphological analyses using Dynamic Light Scattering (DLS) and Scanning Electron Microscopy (SEM). For these experiments, due to the intrinsic limitations of the methods employed, the analysis was performed in culture medium without FBS. The DLS measurements of bare NPs revealed a hydrodynamic mean diameter of 640 ± 16 nm (Figure 5). 

**Figure 6:**
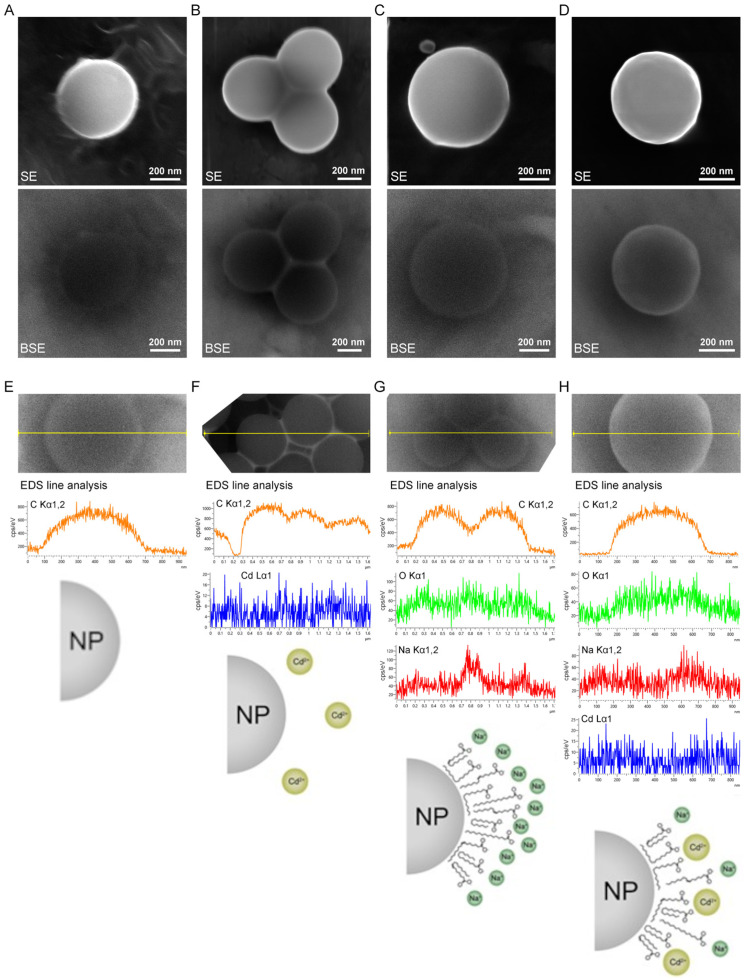
Scanning Electron Microscopy images acquired with secondary electrons (SE, first row) and backscattered electrons (BSE, second row) of (**A**) NPs, (**B**) NPs + Cd, (**C**) NPs + FFA, (**D**) NPs + Cd + FFA. Energy-Dispersive X-ray Spectroscopy line analysis of (**E**) NPs, (**F**) NPs + Cd, (**G**) NPs + FFA, (**H**) NPs + Cd + FFA. Schematics illustrate the interaction between NPs, Cd, and FFA for each condition, visually representing their combined effects.

This result is consistent with SEM evaluations (Figure 6A), which indicated diameters of approximately 500 nm. The addition of cadmium (NPs + Cd) did not significantly affect the hydrodynamic diameters, as evidenced by a mean value of 648 ± 16 nm. This observation likely arises from the minimal interaction between polystyrene NPs and cadmium ions. Cadmium, being a heavier atom compared to carbon, appears brighter in backscattered electron (BSE) analysis and can be easily identified within a carbon-based matrix. In fact, the morphological evaluation from BSE (Figure 6B) and EDS line analysis (Figure 6F) identified cadmium deposits primarily located in the gaps between the NPs. This finding supports the low degree of specific interactions between the NP surfaces and the metal ions; rather, the areas with higher cadmium content are likely a result of solution evaporation and capillary action. Consequently, cadmium is non-homogeneously distributed over the NP surfaces. In contrast, when FFA mixture was added to the NP suspensions (NPs + FFA), an increase in the hydrodynamic diameter was observed, with a mean value of 944 ± 12 nm. This increase can be attributed to interactions between the NP surfaces and the FFA, which likely forms a layer around the NPs. Given the polymeric nature of the NPs, these interactions are hypothesized to be predominantly hydrophobic, with FFA molecules oriented such that their alkyl chains face the NPs and their polar heads, specifically the carboxylic groups, point outward. The EDS line analysis (Figure 6G) indicated that oxygen atoms associated with the carboxylic groups and sodium ions (as counter ions) are evenly distributed over the NP surfaces, as suggested by their higher concentrations detected at the NPs borders. Finally, the addition of cadmium to NPs that were already mixed with FFA (NPs + Cd + FFA) resulted in hydrodynamic diameters measuring 769 ± 23 nm. This value falls between those of bare NPs and NPs mixed with FFA. Given the proposed orientation of FFA when interacting with the NPs, it is reasonable to consider the generation of electrostatic interactions between cadmium ions and the carboxyl groups of FFA, forming an additional external polar layer. In this scenario, cadmium ions, acting as counter ions for FFA, limit the mobility of the due to their large size. Additionally, a decrease in the hydrodynamic diameter of the resulting NPs + Cd + FFA system is observed due to the divalent nature of cadmium ions, which is associated with stronger interactions involving multiple FFA chains. Evidence of FFA surrounding NPs, with cadmium ions uniformly distributed on their surfaces, can be seen in the EDS line analysis (Figure 6H). Additionally, the lower ratio of sodium to oxygen atoms in the case of NPs + Cd + FFA (Na/O = 0.21) compared to NPs + FFA (Na/O = 0.35) supports the hypothesis that sodium ions, being monovalent and smaller compared to cadmium ions, are displaced by divalent cadmium ions, which serve as more stable counter ions to the carboxyl groups of FFA, giving a more compact corona surrounding the NPs.

## 3. Discussion

This study introduces a novel perspective by investigating the impact of polystyrene nanoplastics (NPs) not only as isolated entities but also in the context of their interactions with molecules they might encounter in the environment. These include both ambient contaminants, such as heavy metals, and endogenous biological molecules. Specifically, we examined the effects of NPs on HepG2 cells, focusing on cell viability, ROS production, and lipid content. Unlike previous studies, which predominantly explored the direct cellular effects of polystyrene NPs (rev. in [42]), our work is aimed to assess their interactions with external substances like cadmium, a heavy metal contaminant, and endogenous compounds such as free fatty acids (FFA). This approach reflects real-world scenarios where NPs, with their high surface area and adsorption capacity, might bind to harmful chemicals before or after entering the human body. By simulating these interactions, our study provides deeper insights into how NPs might exacerbate the health risks associated with environmental pollutants, offering a more comprehensive understanding of their potential impact on cellular processes. 

In our study, we employed 500 nm polystyrene NPs, which are likely small enough to be taken up at the intestinal level [10,43]. Additionally, the Peyer’s patches in the ileum can uptake particles in the range of 0.1 to 10 μm via endocytosis, as reviewed by Wright and Kelly [11]. Since substances absorbed in the intestine ultimately reach the liver, hepatocytes are potentially exposed to high concentrations of NPs. We simulated this scenario by studying the possible detrimental effects of NPs exposure on the widely used HepG2 cell model. Consistent with prior studies by Dong et al. [44], da Silva Brito et al. [45] and Peng et al. [46] in similar models, our results indicate that only high concentrations of NPs, far exceeding environmental relevance, lead to significant ROS induction. Despite generating ROS at high concentrations, NPs did not significantly impact cell viability in the conditions we tested. The lack of cytotoxicity provoked by short-term exposure to NPs aligns with the findings of Peng et al. [46] and Banerjee et al. [47]. On the other hand, some studies show that nanoplastics can be cytotoxic [44,48,49] and induce ROS production [45,46,50] in different experimental models. Overall, these seemingly contradictory results suggest that the toxicity of nanoplastics may depend on their type, size, or material composition, and that some cell types are likely more sensitive to the presence of NPs than others. 

In addition to the effects directly linked to cell death, like overt cytotoxicity and ROS production, we aimed to investigate other potential harmful effects of NPs. These effects might not directly cause cell death but could still be harmful. We examined their impact on lipid accumulation, inspired by studies suggesting that exposure to NPs might contribute to obesity and metabolic disorders [20,21,51,52], possibly through mechanisms involving metabolic dysregulation. In our experimental system, we demonstrated that NPs alone neither interfere with intracellular lipid metabolism nor with the internalization of FFA. However, this experimental setup allowed us to uncover an often overlooked aspect and the most innovative result of our work, highlighting that the interaction of NPs with environmental agents can significantly alter their behavior and biological effects. Specifically, we observed that NPs affect lipid uptake, particularly when cadmium is present. This interference exhibits a clear dose-response pattern, with lipid internalization by cells decreasing as NP concentration increases. This effect underscores the capacity of NPs to interact with and sequester biologically essential molecules like lipids. Interestingly, when cadmium is added to the system the hydrodynamic diameter of the NP-FFA complexes decreases, and the related SEM imaging shows the presence of heavy atoms surrounding the NPs. This suggests that cadmium might have an active role in the interaction between NPs and lipids, promoting a tighter and more stable binding. This stabilization effect highlights a complex interplay between NPs, metals, and biological molecules. Measuring the NP hydrodynamic diameters allowed us to demonstrate the formation of a molecular corona under specific conditions. Additionally, changes in the size of the corona were detected based on the nature of the medium and the extent of interactions that occurred. Notably, a size reduction caused by cadmium binding, which stabilizes NP-lipid interactions, was observed. Since lipids are essential for many cellular functions, such as maintaining membrane integrity and storing energy, these interactions could significantly impact cell metabolism and health. Such mechanisms have been suggested in the broader literature on NP-lipid interactions, such as in Yuan et al. [53] where NPs were found to alter lipid membranes. 

The morphological analysis does not allow us to state a direct interaction between NPs and Cd. However, this phenomenon was indirectly observed in cytotoxicity experiments, where NPs likely sequestered Cd and consequently significantly reduced, in a dose-dependent pattern, the cytotoxicity of Cd at concentrations near its LC_50_. Analogous results on the interactions between NPs and heavy metals have been previously reported in studies such as Lian et al. [54], who observed that the presence of polystyrene NPs can reduce cadmium toxicity in wheat, suggesting similar sequestration phenomena. We believe that the apparent discrepancy between cytotoxicity and SEM results can be explained by the different conditions under which the two analyses were conducted. The morphological analysis was performed in culture medium in the absence of serum—a condition necessary to avoid interference with image acquisition—resulting in a very low concentration of amino acids. In contrast, the cytotoxicity experiments, as well as all other cell-based experiments, were conducted in the presence of 10% FBS. Literature evidence shows that in complete culture media, NPs form a protein corona [55,56,57], which facilitates their interaction with ions present in the environment [58,59]. Based on this information, we suggest that the sequestration of cadmium ions by NPs—undetected in the SEM analysis but evident in the cytotoxicity experiments—is facilitated by the formation of a protein corona. This infers that in biological fluids NPs may capture harmful metals, thereby lowering their immediate bioavailability and toxicity to cells. The potential for NPs to alter the chemical form and bioavailability of metals adds a critical dimension to understanding NP ecotoxicity. While the sequestration of cadmium may reduce its acute toxicity, it does not eliminate the risk of eventual metal release in other environments or conditions. These findings offer critical insights into how NPs can serve as carriers for both metals and lipids, potentially altering the bioavailability of essential nutrients and toxic substances simultaneously.

Studies like Rochman et al. [60] and Koelmans et al. [26] support this perspective, highlighting the ability of microplastics to act as vectors for various pollutants, potentially transporting them across ecosystems and releasing them in different environmental contexts. As demonstrated in the literature [61], NPs internalization varies depending on the cell type involved. This reinforces the concern that once internalized, particles can desorb pollutants intracellularly, leading to complex interactions and unpredictable toxicological outcomes, as these effects are influenced not only by the individual and combined toxicity of NPs and pollutants but also by their synergistic, potentiating, or antagonistic behaviors [37].

Overall, these results highlight a critical aspect of microplastic research: the unpredictability of NP behavior in complex environmental and biological matrices and the dual role of microplastics as both passive contaminants and active transporters of environmental pollutants, complicating the prediction of their all-encompassing toxicological profile. While NPs themselves may not be highly toxic at environmentally relevant concentrations, their interactions with other pollutants (such as cadmium) and biological molecules (such as lipids) complicate efforts to model their impact accurately. This study highlights the need for more research into the interactome of NPs. By exploring NPs interactions in more complex environmentally and biologically relevant systems, we can better predict their fate and behavior from a human health perspective, as suggested by Alijagic et al. [37].

## 4. Materials and Methods

### 4.1. Reagents

The 500 nm diameter monodispersed pristine polystyrene beads were obtained from microParticles GmbH (Berlin, Germany).

AdipoRed assay reagent was purchased from Lonza (Walkersville, MD, USA), NucBlue Live ReadyProbes Reagent from Invitrogen (Carlsbad, CA, USA). The CellTiter-Glo^®^ luminescent Cell Viability Assay was purchased from Promega (Madison, WI, USA).

Cadmium (CdCl_2_, catalogue number 202908) was purchased from Sigma-Aldrich (Saint Louis, MO, USA) like all other chemicals unless otherwise specified. 

### 4.2. Cell Cultures

HepG2 human hepatoma cell line (European Collection of Authenticated Cell Cultures ECACC catalogue number 85011430) was purchased from Sigma-Aldrich. Cells were cultured in Minimum Essential Medium Eagle (MEM) supplemented with 10% fetal bovine serum (FBS), 2 mM L-glutamine, 50 IU/mL penicillin, 50 µg/mL streptomycin and 1% non-essential amino acids.

### 4.3. Cell Viability

For the cell viability assays, cells were seeded in black, flat clear bottom, polystyrene 96-well culture plates (Greiner Bio-One, Kremsmünster, Austria), at a density of 3 × 10^4^ cells/well and incubated overnight at 37 °C. The day after, the cells were exposed alternatively to 10–100–1000 µg/mL NPs, to 0.1–100 µM Cd [62], or to NPs (1–100 µg/mL) combined with 50–150 µM Cd. The solutions containing microplastics and the environmental pollutant were prepared, for each experiment, immediately before being brought into contact with the cells. 

Cell viability was assessed after 24 (for NPs, Cd and NPs + Cd) and 48 h (for NPs only) exposure using the CellTiter-Glo^®^ luminescent Cell Viability Assay (Promega, Madison, WI, USA). This test is based on the quantification of ATP, which indicates the presence of metabolically active cells. Briefly, after the period of exposure to the environmental pollutants (alone or in combination) the medium was removed and cells were washed with phosphate-buffered saline (PBS) before adding CellTiter-Glo^®^ reagent diluted 1:1 in PBS; then, after a 10-min dark incubation period at room temperature, the Infinite M Plex microplate reader (Tecan, Männedorf, CH) was used to detect and quantify luminescence, which is directly related to the quantity of viable cells. 

All the experiments were repeated at least three times, and 8 wells were devoted to each exposure condition in each experiment.

### 4.4. Reactive Oxygen Species (ROS) Production

HepG2 cells were grown in black 96-well microplates with a clear bottom (Greiner Bio-One, Kremsmünster, Austria). After 24 h seeding, they were exposed to the stressor menadione (MEN) as a positive control (20 µM), or to different concentrations of NPs (1–100 µg/mL) for 1 h. During the last 30 min of the stimulation, cells were loaded in the dark with 5 µM of CellROX^®^ Green Reagent, a probe that exhibits bright green fluorescence upon oxidation by ROS. At the end of the incubation period cells were washed twice with PBS, and fluorescence was acquired with the Infinite M Plex microplate reader (Tecan, Männedorf, CH) at excitation/emission of 485/535 nm. Data were expressed as percentages of fluorescence referring to the control condition; the percentage values of the three experiments were then analyzed to calculate mean ± standard deviation (SD).

### 4.5. In Vitro Steatosis Induction and Lipid Quantification

For steatosis induction experiments, the free fatty acid mixture was prepared by coupling sodium palmitate (Na+-hexadecanoate) and sodium oleate (Na+-(Z)-octadec-9-enoate) (1:2 ratio) with 1% *w*/*v* FFA-free BSA in serum free MEM, at 38 °C in agitation overnight, to allow FFA coupling with BSA; the mixture was then filtered and used immediately in subsequent experiments or frozen at 20 °C. Control cells were grown with serum-free MEM containing 1% *w*/*v* BSA. 

For each experiment, 3 × 10^4^ cells/well were seeded in 96-well black clear bottom plates (Greiner Bio-One, Frickenhausen, Germany). To evaluate the impact of NPs and Cd on endogenous (no FFA addition) and internalized (with FFA addition) lipid content in hepatic cells, the following experimental setup was employed (Table 1):

**Table 1:** Graphical representation of the experimental schemes adopted to evaluate the impact of polystyrene nanoplastics (NPs) and cadmium (Cd) on endogenous (1) and FFA-induced (2 and 3) lipid content in HepG2 cells. A/N = adipored-nucblue staining for lipid quantification.

	0–24 h	24–48 h	48–72 h
1	NPs + Cd → A/N		
2	starving	FFA	NPs + Cd → A/N
3	starving	NPs + Cd + FFA → A/N	

1. to assess the effects of NPs and Cd on endogenous lipids, cells were exposed for 24 h to these agents, followed by A/N assay.

2. to investigate NPs and Cd effects on steatotic HepG2, cells underwent 24-h starvation followed by 24 h exposure to FFA. Subsequently, cells were exposed to NPs and Cd for the next 24 h, followed by A/N assay.

3. to examine the impact of NPs + Cd exposure during lipid accumulation, cells underwent 24-h starvation then were simultaneously exposed to FFA, NPs, and Cd, followed by A/N assay.

At the end of the experiments, the medium was removed, and cells were rinsed with PBS before replacing with 200 μL/well of a live-cell dye mixture containing AdipoRed™ Assay Reagent (triglyceride accumulation measurement; Lonza, Basel, Switzerland) and NucBlue^®^ Live ReadyProbes^®^ Reagent (cell proliferation/cytotoxicity measure of DNA content; (Thermo Fisher Scientific, Waltham, MA, USA) (25 μL and 1 drop, respectively, for each mL of PBS). Plates were protected from light and incubated at room temperature for approximately 40 min. Fluorescence was then measured with Infinite M plex microplate reader (Tecan, Männedorf, Switzerland); for AdipoRed, quantification excitation was performed at 485 nm and emission read at 535 nm, while for NucBlue, excitation was at 360 nm and emission read at 460 nm. The ratio between the AdipoRed and NucBlue (A/N) values for each well is a key metric that allows quantification of the lipid content per cell, effectively normalizing for the number of cells present in each well. This approach ensures that lipid measurements are accurately scaled to individual cell counts. 

### 4.6. Dynamic Light Scattering (DLS)

The nanoparticle dispersions were prepared in MEM and, immediately before performing the analysis were subsequently diluted 1:10 with ultrapure water to ensure reliable dynamic light scattering measurements. The evaluation of hydrodynamic diameter was conducted using dynamic light scattering with a Malvern Zetasizer Nano ZS instrument (Malvern, UK). For each sample, triplicate measurements were performed, with each measurement averaging at least 10 runs at a temperature of 25 °C. 

### 4.7. Scanning Electron Microscopy (SEM)

The morphology was examined using scanning electron microscopy (SEM). To minimize interferences, the nanoparticles were dispersed in culture medium (MEM) as for the in-vitro tests, excluding the addition of FBS which would have interfered with the sample preparation and image acquisition. The dispersions were then transferred onto a SEM stub and allowed to dry overnight at room temperature. Images were captured with a FE-SEM Tescan S9000G (Brno, Czech Republic), equipped with EDS microanalysis from Oxford AZTEC (Abingdon-on-Thames, UK). Secondary (SE) and backscattered (BSE) electrons at an accelerating voltage of 7 kV were used, under ultrahigh resolution conditions. EDS line sum spectra were obtained to analyze the chemical composition of the nanoparticles.

### 4.8. Statistical Analysis

All statistical analyses were performed using GraphPad Prism version 9.0 (GraphPad Software, LLC, Boston, MA, USA). Data are presented as the means ± SDs of at least three experiments performed in triplicate. Statistically significant differences between treatment and control groups were assessed by a one-way analysis of variance (ANOVA) followed by Tukey’s multiple-comparison post hoc test. Differences were considered to be statistically significant for *p* < 0.05.

## 5. Conclusions

In conclusion, this study demonstrates that while the direct toxic effects of polystyrene nanoplastics on cellular systems can be minimal at environmentally relevant concentrations, their capacity to interact with other environmental contaminants and biological molecules adds significant complexity to their potential ecological and toxicological impacts. These findings underscore the importance of considering not only the direct effects of NPs but also their role as carriers and modifiers of environmental pollutants behavior. The ecological implications of NPs highlight the need for stricter environmental regulations to mitigate their accumulation in ecosystems. Understanding their interactions with environmental pollutants and biological systems can inform policies aimed at reducing NP release, promoting safer waste management practices, and protecting biodiversity. Future environmental risk assessments of microplastics should incorporate molecular interactions to provide a more comprehensive understanding of their potential harm, while policies should focus on monitoring, risk assessment, and the development of sustainable materials to limit NP exposure.

This article is a revised and expanded version of a poster presented at Eurotox 2024, Copenhagen, Denmark, 8–11 September 2024 [63].

## Data Availability

The raw data supporting the conclusions of this article will be made available by the authors on request.
